# Adjunctive corticosteroids may be associated with better outcome for non-HIV *Pneumocystis* pneumonia with respiratory failure: a systemic review and meta-analysis of observational studies

**DOI:** 10.1186/s13613-020-00649-9

**Published:** 2020-03-20

**Authors:** Lin Ding, Huixue Huang, Heyan Wang, Hangyong He

**Affiliations:** 1 Department of Respiratory and Critical Care Medicine, Beijing Luhe Hospital, Beijing, China; 2 Department of Medicine, Beijing University of Technology Hospital, Beijing, China; 3 Department of Critical Care Medicine, The Sixth Hospital of Guiyang, Guiyang, Guizhou China; 4 Beijing Key Laboratory of Respiratory and Pulmonary Circulation Disorders, Beijing, China; 5 Beijing Engineering Research Center for Diagnosis and Treatment of Pulmonary and Critical Care, Beijing, China; 6 Department of Respiratory and Critical Care Medicine, Beijing Institute of Respiratory Medicine, Beijing Chao-Yang Hospital, Capital Medical University, No. 8 Gongren Tiyuchang Nanlu, Chaoyang District, Beijing, 100020 China

**Keywords:** Corticosteroids adjunctive treatment (CAT), *Pneumocystis* pneumonia (PCP), Non-HIV, Respiratory failure

## Abstract

**Background:**

Evidence supporting corticosteroids adjunctive treatment (CAT) for *Pneumocystis jirovecii* pneumonia (PCP) in non-HIV patients is highly controversial. We aimed to systematically review the literature and perform a meta-analysis of available data relating to the effect of CAT on mortality of PCP in non-HIV patients.

**Methods:**

We searched Pubmed, Medline, Embase, and Cochrane database from 1989 through 2019. Data on clinical outcomes from non-HIV PCP were extracted with a standardized instrument. Heterogeneity was assessed with the *I*^2^ index. Pooled odds ratios and 95% confidence interval were calculated using a fixed effects model. We analyzed the impact of CAT on mortality of non-HIV PCP in the whole PCP population, those who had hypoxemia (PaO_2_ < 70 mmHg) and who had respiratory failure (PaO_2_ < 60 mmHg).

**Results:**

In total, 259 articles were identified, and 2518 cases from 16 retrospective observational studies were included. In all non-HIV PCP cases included, there was an association between CAT and increased mortality (odds ratio, 1.37; 95% confidence interval 1.07–1.75; *P* = 0.01). CAT showed a probable benefit of decreasing mortality in hypoxemic non-HIV PCP patients (odds ratio, 0.69; 95% confidence interval 0.47–1.01; *P* = 0.05). Furthermore, in a subgroup analysis, CAT showed a significantly lower mortality in non-HIV PCP patients with respiratory failure compared to no CAT (odds ratio, 0.63; 95% confidence interval 0.41–0.95; *P* = 0.03).

**Conclusions:**

Our meta-analysis suggests that among non-HIV PCP patients with respiratory failure, CAT use may be associated with better clinical outcomes, and it may be associated with increased mortality in unselected non-HIV PCP population. Clinical trials are needed to compare CAT vs no-CAT in non-HIV PCP patients with respiratory failure. Furthermore, CAT use should be withheld in non-HIV PCP patients without hypoxemia.

## Introduction

*Pneumocystis jirovecii* pneumonia (PCP) is a major cause of acute respiratory failure and death in immunocompromised patients. Malignant disease with chemotherapy, corticosteroids treatment for inflammatory autoimmune diseases, and transplantation of solid organs or bone marrow are the leading causes of T cell suppression, which is associated with a high risk of opportunistic infections, including PCP [1, 2]. The mortality associated with PCP in the non-HIV population group remains high, ranging from 19 to 76% [3–5], despite the availability of effective antimicrobial agents such as trimethoprim/sulfamethoxazole (TMP-SMZ) and pentamidine.

Early studies done in HIV-positive patients showed that corticosteroids adjunctive treatment (CAT) was associated with a dramatic decrease in mortality during PCP episodes [6, 7]. The pathophysiology of PCP may differ between patients with and without HIV infection. Conceivably, these differences between the two populations might affect the ability of steroids therapy to provide therapeutic benefits [8].

Alternative therapies such as the use of adjunctive agents in an effort to reduce the mortality associated with non-HIV population have been evaluated by several retrospective studies. Unfortunately, the data for CAT among the non-HIV population are highly controversial [5, 9], and one study even indicated potential harm with CAT in severe cases admitted into the intensive care unit (ICU) [10].

Based on relatively limited and controversial data from studies available at this stage, the primary objective of this investigation was to systematically review the literature and perform a meta-analysis of available data relating to evaluation of whether CAT can reduce mortality in non-HIV patients with PCP.

## Methods

### Data source and study selection

A literature search of Pubmed, Medline, Embase, and Cochrane database from 1989 to September 2019 was performed to find published articles evaluating adjunctive corticosteroids therapy for patients with non-HIV PCP. We limited studies to human subjects and searched for the following terms: (Pneumocysti*[text word] OR PCP[text word] OR “*Pneumocystis* Infections”[MESH] OR “*Pneumocystis jirovecii*”[MESH] OR “Pneumonia, *Pneumocystis*”[MESH] OR “*Pneumocystis* carinii”[MESH]) AND (treat* [text word] OR adjunct* [text word] OR treatment* [text word] OR steroid*[text word] OR corticosteroid*[text word] OR glucocorticoid*[text word] OR “Glucocorticoids”[MESH] OR “Adrenal Cortex Hormones”[MESH] OR “Steroids”[MESH]) NOT ((“animals”[MeSH] NOT “humans”[MeSH]) AND (“non-HIV” OR “non-AIDS” OR “non-HIV-infected” OR “HIV-uninfected” OR “AIDS uninfected” OR “HIV-negative”)). In addition, we also reviewed the references listed in the identified articles and performed a manual search of the related articles to identify all relevant and eligible articles and to minimize publication bias.

Two researchers screened and evaluated the eligibility of all studies independently, and a third reviewer intervened whenever there was a disagreement. The inclusion criteria were (1) original research report of PCP in non-HIV patients; (2) report of mortality from patients with non-HIV PCP; (3) reported with data on mortality between patients treated with and without adjunctive corticosteroids, and (4) written in English. The exclusion criteria were (1) written in languages other than English; (2) not related to PCP pneumonia; (3) not an original research; (4) in vitro studies; (5) reports of single case experiences. The intervention of interest was corticosteroids adjunctive therapy. The comparison groups were patients treated for non-HIV PCP with and without adjunctive corticosteroids. The outcome of interest was mortality in all non-HIV PCP patients and mortality in those with acute hypoxemic respiratory failure, as defined by the study investigators. The meta-analysis of observational studies in epidemiology criteria were used to conduct this investigation, and the article follows MOOSE criteria [11].

### Data extraction

Two investigators extracted data independently from each eligible study, using a standardized data extraction form. Discrepancies in data extraction underwent arbitration by a third reviewer, and consensus was obtained by verbal discussion. Data collected from each study included author, year of study, country, age group of patients, number of patients, causes of immunosuppression, method of PCP diagnosis, treatment of PCP, definition of adjunctive corticosteroid use, dose of steroids used, and mortality.

### Data synthesis and data analysis

Data on non-HIV PCP outcomes were collected from all articles. All-cause mortality and clinical cure rates, as defined by the individual studies, were the primary outcome measures used in this meta-analysis. Odds ratios for mortality were calculated for each article. The significance level for this meta-analysis model was set at *P* < 0.05. Forest plots were provided for mortality in this meta-analysis. We used the Q statistic to test the existence of heterogeneity; a *P* value of less than 0.10 was considered significant for heterogeneity. *I*^2^ was employed to assess the proportion of total variability due to heterogeneity. An *I*^2^ value of approximately 25% was regarded as low heterogeneity, 50% as medium, and 75% as high heterogeneity. Publication bias was assessed and an applicability concerns graph/summary was conducted using Review Manager version 5.3 (Cochrane Collaboration, Oxford, UK).

For the mortality in all non-HIV PCP patients, we performed a subgroup meta-analysis on different types of mortality reported (ICU mortality, hospital mortality, 30-day mortality and 60-day mortality). For the mortality in patients with hypoxemia or respiratory failure, we performed a subgroup meta-analysis on different severity of hypoxemia: hypoxemia patients with a PaO_2_ < 70 mmHg (including patients with 60 mmHg < PaO_2_ < 70 mmHg and patients with PaO_2_ < 60 mmHg), and respiratory failure patients with a PaO_2_ < 60 mmHg. For the different doses of CAT used, we performed a subgroup meta-analysis on different patients with high dose (> 240 mg prednisone equivalent) or low dose (1 mg/kg/day prednisone equivalent) CAT.

## Results

### Literature search

**Fig. 1:**
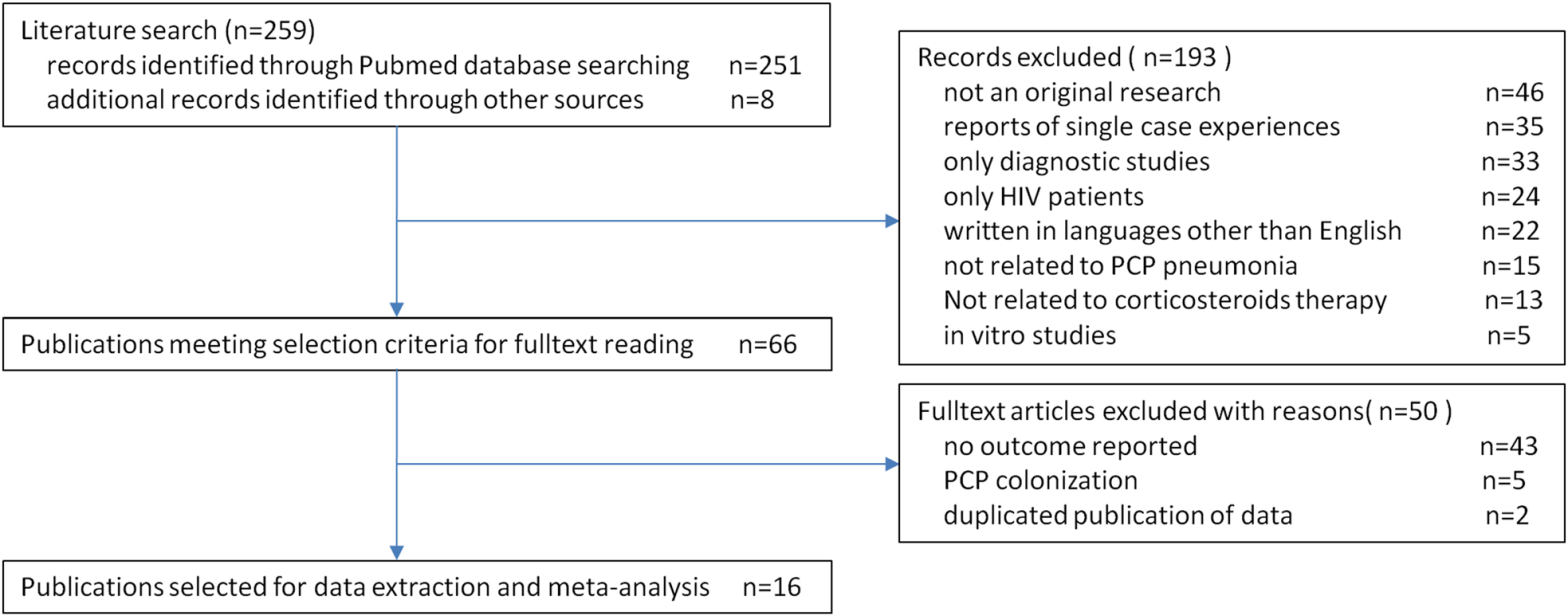
Flowchart of study selection, process and reasons for exclusion of references

The literature search yield a total of 259 articles possibly related to non-HIV PCP with CAT. By reviewing the titles and abstracts, studies were mainly excluded due to the following: not an original research (*n* = 46); reports of single case experiences (*n* = 35); only diagnostic studies (*n* = 33); only HIV patients (*n* = 24); written in languages other than English (*n* = 22); not related to PCP pneumonia(*n* = 15); not related to corticosteroids therapy (*n* = 13); in vivo/in vitro studies (*n* = 5). After the initial screening, 66 articles with data on treatment of non-HIV PCP remained for full-text review. Of these, 50 articles were excluded from further analysis because they did not include outcome data (*n* = 43), reports of PCP colonization without evidence of infection (*n* = 5), or duplicated reports (*n* = 2). In the final analysis, 16 retrospective cohort investigations of patients treated with CAT were included [3–5, 9, 12–23] (Fig. 1).

### Study characteristics

Demographic variables of the included studies are summarized in Table 1. A total of 16 studies involving 2518 patients were included in this meta-analysis. The 16 studies were conducted in 7 countries as France (*n* = 4), Japan (*n* = 4), United States (*n* = 2), China (*n* = 2), Korea (*n* = 2), Italy (*n* = 1), and Greece (*n* = 1).

**Table 1:** Studies characteristics included in this meta-analysis

Series no.	References author, year of publish	Study design	Country	Total no. of patients	Causes of immunosuppression	Method of PCP diagnosis	Definition of adjunctive corticosteroids use
1	Pareja, 1998	Retrospective single center	USA	30	Organ transplantation = 13; longterm immunosuppressive therapy = 9; Chemotherapy for malignancy = 8	Morphological	≥ 60 mg prednisone daily equivalent
2	Delclaux, 1999	Retrospective two center	France	31	Hematologic malignancy = 24; vasculitis = 3; solid tumor = 2; other = 2	Morphological (demonstration of typical organism in specimens BALF)	1. De novo therapy (for patients who did not receive previous CS): oral prednisone > 1 mg/kg/day or iv methylprednisolone 240 mg for 3 days- > 120 mg for 3 days- > 60 mg for 3 days or longer 2. Rescue therapy(for patients already receiving CS): corticosteroid increased at least 300% of the baseline
3	Zahar, 2002	Retrospective two center	France	39	Malignancy: hematological malignancies = 28 Solid tumor = 7; nonmalignant hematological diseases = 4	Positive results for cytological tests or direct fluorescent antibody staining for PCP in sputum or BALF	240 mg/day for 3 days, with the dosage then tapered during the next 10 days, until no therapy was given
4	Pagano, 2002	Retrospective multicenter	Italy	55	Malignant hematological diseases: on-Hodgkin’s lymphoma = 18 Acute lymphoblastic leukemia = 10 Acute myeloid leukemia = 8; chronic myeloid leukemia = 5	Microbiologically (positive visualization, mAbs, or PCR of PC in sputum or BALF) and/or histologically documented	Methylprednisolone (50–80 mg/day)
5	Roblot, 2003	Retrospective multicenter	France	60	Hematologic malignancies: non-Hodgkin’s malignant lymphoma = 18 Chronic lymphocytic leukemia = 13; acute leukemia = 10 Multiple myeloma = 5; Waldenstro¨m’s diseases = 4; chronic myeloid leukemia = 4 Myelodysplasia = 3; Hodgkin’s diseases = 2; thrombopenia = 1	Microbiological evidence of PC in BALF (silver and eosin þ methylene blue stainings and/or immunostaining using monoclonal antibodies)	Dose and duration of adjunctive corticosteroids use was not detailed reported
6	Bollee, 2007	Retrospective single center	France	56	Cancer: hematologic malignancies = 44; solid tumors = 12	PCP with Giemsa staining and/or immunofluorescence with human monoclonal anti-Pneumocystis cyst antibodies in BALF or sputum	1 mg/kg of prednisone (or equivalent) per day or in a 30-mg increase in the daily prednisone dosage
7	Aoki, 2009	Retrospective single center	Japan	25	Collagen vascular diseases	Elevated concentration of plasma 1,3-d-glucan, and positive PCR test for PCP in sputum	Dose and duration of adjunctive corticosteroids use was not detailed reported
8	Matsumura, 2011	Retrospective multicenter	Japan	82	Inflammatory diseases = 50; solid malignancies = 17 Hematological malignancies = 12; transplantations = 6	Diagnosed with PCP by PCR	Dose and duration of adjunctive corticosteroids use was not detailed reported
9	Moon, 2011	Retrospective single center	Korea	88	Solid-organ transplant = 26; hematologic malignancy = 26 Non-hematologic malignancy = 12; interstitial lung disease = 9 Connective tissue disease = 7; others = 8	Diagnosis was based on a positive result in the direct immunofluorescence assay for PCP	At least 40 mg prednisone twice daily for 5 days
10	Kim, 2014	Retrospective multicenter	Korea	173	Hematologic malignancy = 81; solid tumors = 38 Organ transplantations = 17; others = 37	Positive direct fluorescent antibody (DFA) or PCR results from a sputum or BAL fluid sample	Dose and duration of adjunctive corticosteroids use was not detailed reported
11	Kofteridis, 2014	Retrospective single center	Greece	62	Malignant hematological disease = 31; solid tumor = 16 Chronic inflammatory disease = 15	Positive results of direct fluorescent antibody staining for PCP in samples of induced sputum or BALF	Dose and duration of adjunctive corticosteroids use was not detailed reported
12	Weng, 2016	Retrospective multicenter	China	82	CTD = 65; organ transplant = 3; hematologic malignancy = 3; solid tumor = 4	PCR or methenamine silver stain of samples from BALF, aspirate or sputum	Dose and duration of adjunctive corticosteroids use was not detailed reported
13	Kotani, 2017	retrospective single center	Japan	20	Rheumatoid arthritis or CTD = 9; renal transplant = 7; other = 4	By fluorescent antibody staining using induced sputum or BALF	Methylprednisolone was dependent on the prior dosage of steroids and any underlying diseases
14	Wieruszewski, 2018	Retrospective single center	USA	332	Hematologic malignancy = 162; bone marrow transplantation = 26 Inflammatory of connective tissue disease = 100 Solid organ tumor = 61; solid organ transplantation = 20	A Positive single-copy PCP PCR or smear from a respiratory specimen	Corticosteroids within 48 h
15	Inoue, 2019	Retrospective multicenter	Japan	1299	After transplantation = 34; solid tumor = 245 Malignancy of lymphoid tissue = 160; rheumatoid arthritis = 257 Connective tissue disease = 80	Based on records	Adjunctive corticosteroids were defined < 250 mg/day corticosteroids or high dose pulse steroid therapy which refers to the administration of 250 mg prednisone equivalent a day for one or a few days (usually less than 5 days)
16	Liu, 2019	Retrospective single center	China	84	Hematologic malignancy = 29; autoimmune diseases = 21 Solid cancers = 17; solid organ transplantation = 7	Quantitative real-time PCR	Adjunctive steroids were high dose (≥ 1 mg/kg/day)

BALF, bronchoalveolar lavage fluid; PC, Pneumocystis; PCP, *Pneumocystis* pneumonia; *DFA* direct fluorescent antibody; *PCR* polymerase chain reaction

The type of immunocompromised diseases of non-HIV patient population included hematological malignancies (*n* = 715), solid tumor (*n* = 421), rheumatoid arthritis (*n* = 257), solid organ transplantation (n = 133), and other inflammatory autoimmune diseases (*n* = 393, including collagen vascular disease, interstitial lung disease, connective tissue disease) (Table 1).

Diagnosis of PCP was based on methenamine silver stain under microscopic examination (7 studies), fluorescent antibody staining (8 studies), or polymerase chain reaction (4 studies) of the patients’ sputum/bronchoalveolar lavage fluid, or transbronchial biopsy (1 study), or elevated concentration of plasma 1,3-d-glucan (1 study) (Table 1).

The included investigations differed in their definition of CAT from different centers (shown in Table 1). The investigations defined CAT use as prednisone doses greater than 60 mg/day or methylprednisolone 50–80 mg/day, pulse corticosteroids(high dose corticosteroids as 240 mg/day for 3 days, with the dosage then tapered during the next 10 days, until no therapy was given), prednisone doses of 80 mg or greater, and prednisone doses > 1 mg/kg.

Studies also differed in their definition of mortality (Table 2). Six investigations used in-hospital mortality rates, six investigations used 30 day all-cause mortality, two used 60 day all-cause mortality, and two used ICU mortality rates.

**Table 2:** Studies included in this meta-analysis of the impact of adjunctive corticosteroids on mortality of non-HIV PCP

Series no.	References author, year of publish	Study design	Country	Hospital or centers	Study period	Total no. of patients	Major outcome reported	Mortality	No CAT mortality	CAT mortality	Cases of hypoxemic respiratory dysfunction (hypoxemia: PaO_2_ < 70 mmHg on room air; respiratory failure: PaO_2_ ≤ 60 mmHg on room air)	Mortality in respiratory failure PCP	Mortality in hypoxic PCP and no CAT	Mortality in hypoxic PCP and with CAT
1	Pareja, 1998	Retrospective single center	USA	1 tertiary care urban teaching hospital	1989–1995	30	In-hospital mortality	12/30 (40%)	5/14 (36%)	7/16 (44%)	21/30 (70%, hypoxemia)	12/21 (57.1%)	4/9 (44.4%)	8/12 (66.7%)
2	Delclaux, 1999	Retrospective two center	France	2 medical ICUs	1988–1996	31	ICU mortality	13/31 (42%)	4/8 (50%)	9/23 (39%)	31/31 (100%, respiratory failure)	13/31 (42%)	4/8 (50%)	9/23 (39%)
3	Zahar, 2002	Retrospective two center	France	2 medical ICUs	1989–1999	39	30-day mortality	13/39 (33%)	3/8 (38%)	10/31 (32%)	39/39 (100%, respiratory failure)	13/3 (33%)	3/8 (38%)	10/31 (32%)
4	Pagano, 2002	Retrospective multicenter	Italy	52 hematology divisions	1990–1999	55	30-day mortality	20/55 (36%)	12/33 (36%)	8/22 (36%)	39/55 (71%, hypoxemia)	N/A	N/A	N/A
5	Roblot, 2003	Retrospective multicenter	France	11 infectious diseases units	1995–1999	60	30-day mortality	20/60 (33%)	5/25 (20%)	15/35 (43%)	N/A	N/A	N/A	N/A
6	Bollee, 2007	Retrospective single center	France	1 adult cancer center	2001–2006	56	In-hospital mortality	11/56 (20%)	9/35 (26%)	2/21 (10%)	56/56 (100%, hypoxemia)	11/56 (20%)	9/35 (26%)	2/21 (10%)
7	Aoki, 2009	Retrospective single center	Japan	1 university hospital	2000–2007	25	In-hospital mortality	11/25 (44%)	4/12 (33%)	7/13 (54%)	25/25 (100%, hypoxemia)	11/25 (44%)	4/12 (33%)	7/13 (54%)
8	Matsumura, 2011	Retrospective multicenter	Japan	2 university hospital	2005–2010	82	30-day mortality	20/82 (24%)	4/22 (18%)	16/60 (27%)	57/82 (70%, hypoxemia)	18/57 (32%)	N/A	N/A
9	Moon, 2011	Retrospective single center	Korea	1 university hospital	2007–2010	88	30-day mortality	28/88 (32%)	10/29 (35%)	18/59 (31%)	54/88 (61%, respiratory failure)	28/54 (52%)	10/17 (59%)	18/37 (49%)
10	Kim, 2014	Retrospective multicenter	Korea	2 tertiary care university hospitals	2004–2011	173	In-hospital mortality	62/173 (36%)	0/21 (0%)	62/152 (41%)	88/173 (51%, respiratory failure)	N/A	N/A	N/A
11	Kofteridis, 2014	Retrospective single center	Greece	1 university hospital	2004–2013	62	In-hospital mortality	18/62 (29%)	3/12 (25%)	15/50 (30%)	12/62 (19%, hypoxemia)	N/A	N/A	N/A
12	Weng, 2016	Retrospective multicenter	China	4 ICUs from 2 centers	2012–2015	82	ICU mortality	62/82 (76%)	14/18 (78%)	48/64 (75%)	82/82 (100%, respiratory failure)	62/82 (76%)	14/18 (78%)	48/64 (75%)
13	Kotani, 2017	Retrospective single center	Japan	1 ICU	2008–2012	20	In-hospital mortality	4/20 (20%)	1/11 (9%)	3/9 (33%)	N/A	N/A	N/A	N/A
14	Wieruszewski, 2018	Retrospective single center	USA	1 teaching hospital	2006–2016	332	30-day mortality	74/332 (23%)	6/74 (9%)	68/258 (26%)	N/A	N/A	N/A	N/A
15	Inoue, 2019	Retrospective multicenter	Japan	Nationwide records study	2010–2016	1299	60-day mortality	247/1299 (19%)	34/159 (21%)	213/1140 (19%)	737/1299 (57%, respiratory failure)	189/737 (26%)	26/71 (37%)	163/661 (25%)
16	Liu, 2019	Retrospective single center	China	1 medical center	2015–2016	84	60-day mortality	39/84 (46%)	16/41 (39%)	23/43 (53%)	57/84 (68%, respiratory failure)	N/A	N/A	N/A
Total	1998 ~ 2019	16 retrospective 8 single center 8 multi center	From 7 countries	1 nationwide data 8 hospital wide data 4 ICU wide data 3 ward wide data	1989–2016	2518	2 for ICU mortality 6 for in-hospital mortality 6 for 30-day mortality 2 for 60-day mortality	654/2518 (26%)	130/522 (25%)	524/1996 (26%)	6 with hypoxemia (3 with CAT use reported) 7 with respiratory failure (5 with CAT use reported)	339/1040 (33%)	74/178 (42%)	265/862 (31%)

CAT, corticosteroids adjunctive therapy; ICU, intensive care unit; N/A, not available; PCP, *Pneumocystis* pneumonia

### Impact of corticosteroids adjunctive therapy on mortality of non-HIV PCP

**Fig. 2:**
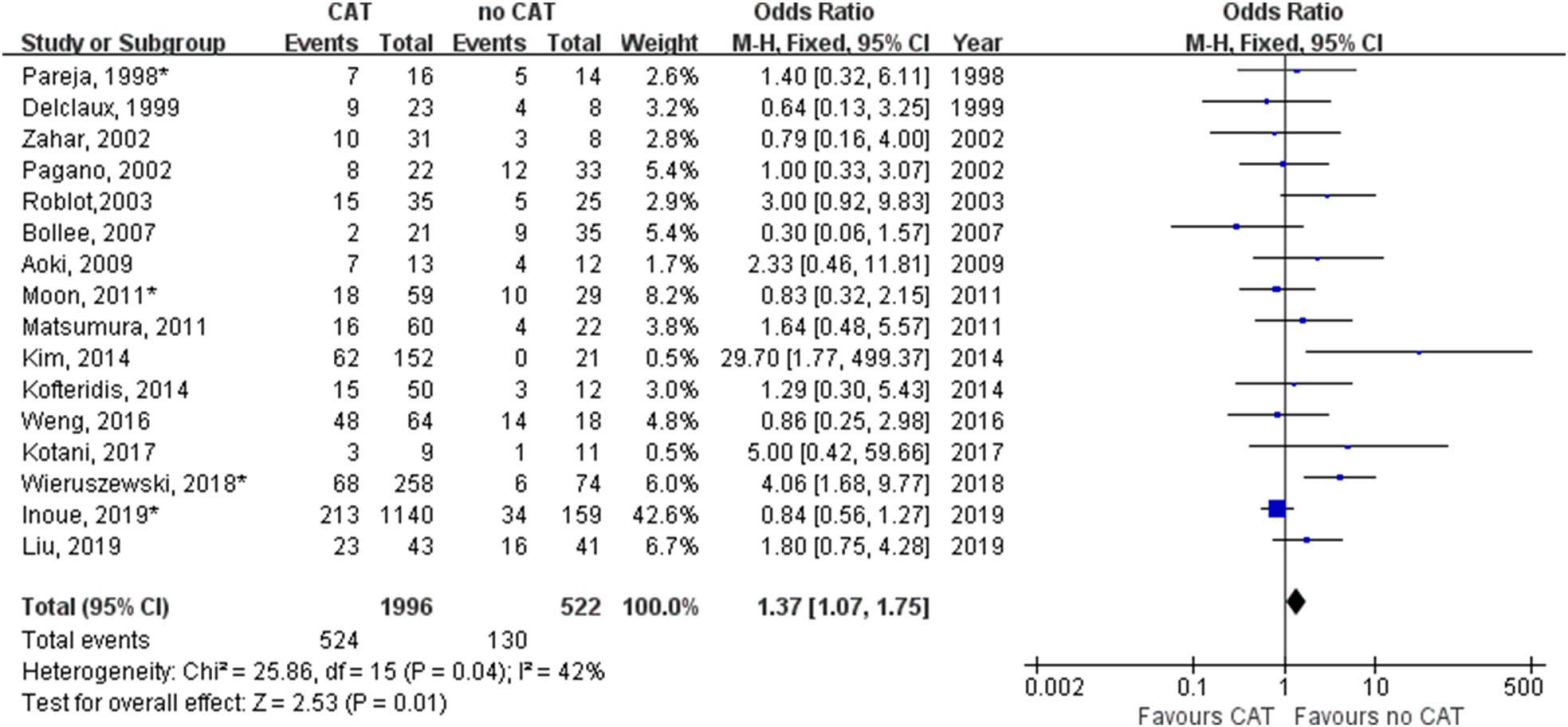
Forest plot for effect of corticosteroids adjunctive therapy (CAT) on mortality of non-HIV PCP

We performed a meta-analysis on mortality associated with or without CAT in 16 studies in all non-HIV PCP patients (Table 2). Figure 2 shows treatment with CAT for non-HIV PCP patients have an significant impact on mortality (odds ratio, 1.37; 95% confidence interval 1.07–1.75; *P* = 0.01). The CAT was related with a higher mortality in all non-HIV PCP patients included.

Subgroup analysis for different types of mortality were performed (as shown in Additional file 1: Figure 2.2). Treatment with CAT for non-HIV PCP patients had no significant impact on ICU mortality (odds ratio, 0.77; 95% confidence interval 0.29–2.08; *P* = 0.61, Additional file 1: Figure 2.2.1) and 60-day mortality (odds ratio, 0.97; 95% confidence interval 0.67–1.41; *P* = 0.89, Additional file 1: Figure 2.2.4). Treatment with CAT for non-HIV PCP patients had a significant impact on hospital mortality (odds ratio, 2.14; 95% confidence interval 1.17–2.91; *P* = 0.01, Additional file 1: Figure 2.2.2) and 30-day mortality (odds ratio, 1.85; 95% confidence interval 1.20–2.84; *P* = 0.005, Additional file 1: Figure 2.2.3).

### Impact of corticosteroids adjunctive therapy on mortality of non-HIV PCP with hypoxemia or respiratory failure

**Fig. 3:**
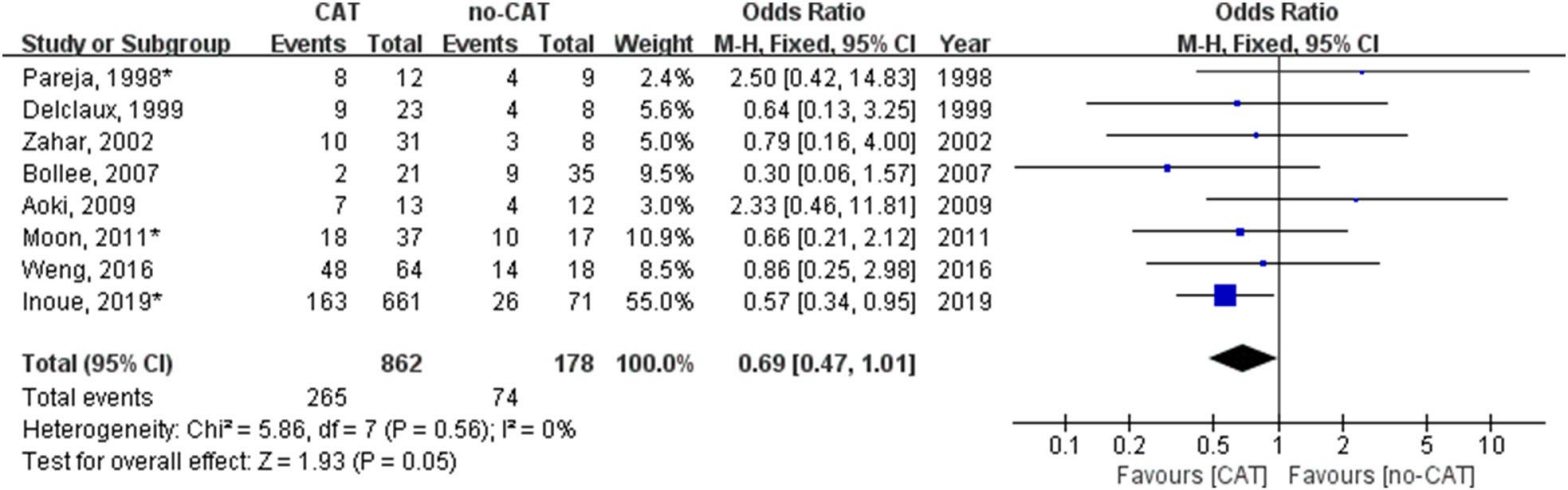
Forest plot for effect of corticosteroids adjunctive therapy (CAT) on mortality of non-HIV PCP with acute hypoxemic respiratory failure

We performed another meta-analysis on mortality associated with or without CAT in 8 studies in which non-HIV PCP patients with hypoxemia or acute hypoxemic respiratory failure were reported (Table 2). Figure 3 shows CAT was related to a trend of lower mortality for non-HIV PCP patients with hypoxemia (odds ratio, 0.69; 95% confidence interval 0.47–1.01; *P* = 0.05).

**Fig. 4:**
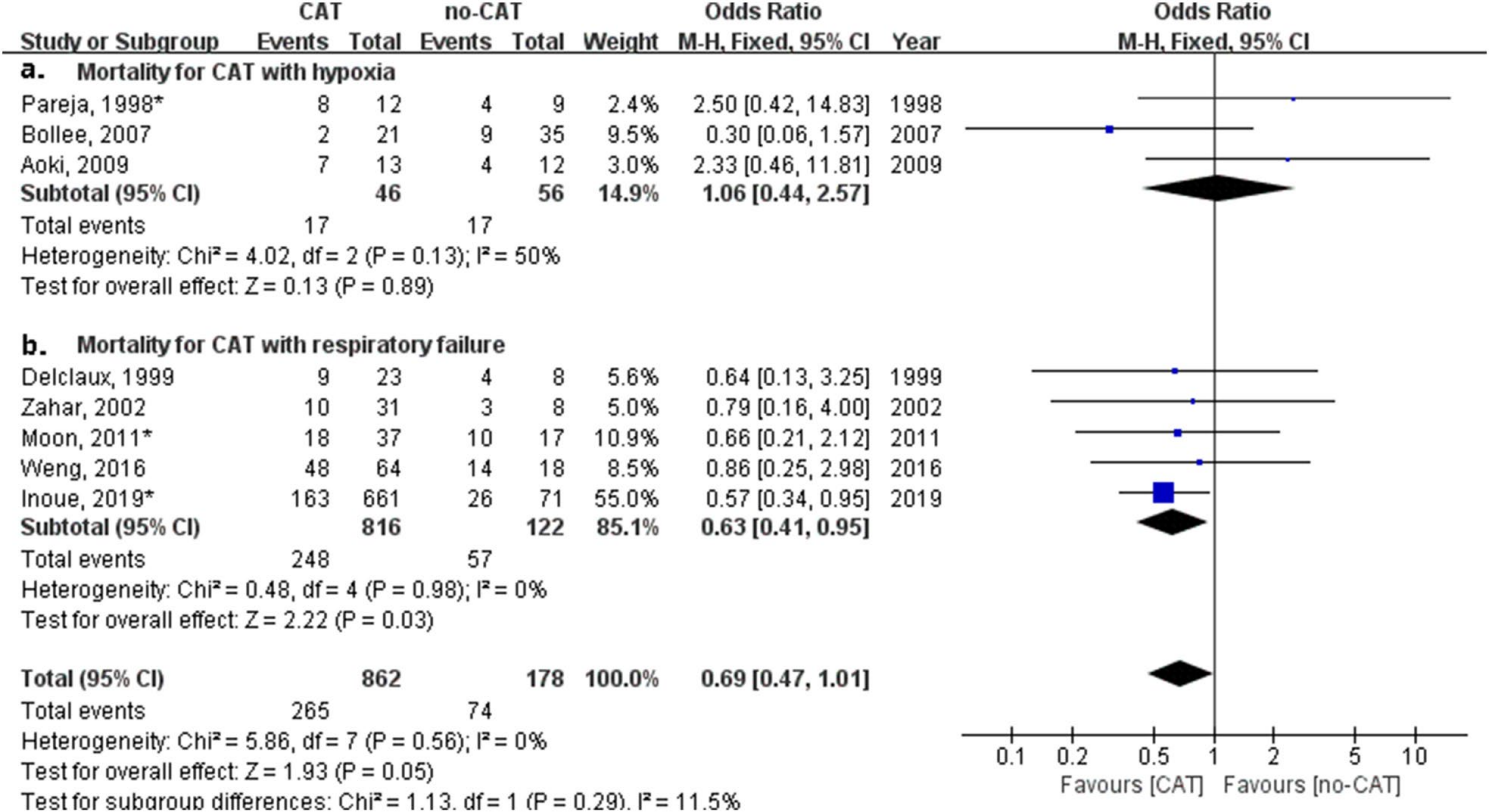
Forest plot of subgroup analysis for effect of corticosteroids adjunctive therapy (CAT) on mortality of non-HIV PCP with hypoxemia or respiratory failure

Subgroup analysis for hypoxemia or respiratory failure was performed (as shown in Fig. 4). Treatment with CAT for non-HIV PCP patients with hypoxemia had no significant impact on mortality (odds ratio, 1.06; 95% confidence interval 0.44–2.57; *P* = 0.89, Fig. 4a). Treatment with CAT for non-HIV PCP patients with respiratory failure had a significant impact on mortality (odds ratio, 0.63; 95% confidence interval 0.41–0.95; *P* = 0.03, Fig. 4b). CAT was related to a significantly lower mortality for non-HIV PCP patients with respiratory failure.

### Impact of high- or low-dose corticosteroids adjunctive therapy on mortality of non-HIV PCP

**Fig. 5:**
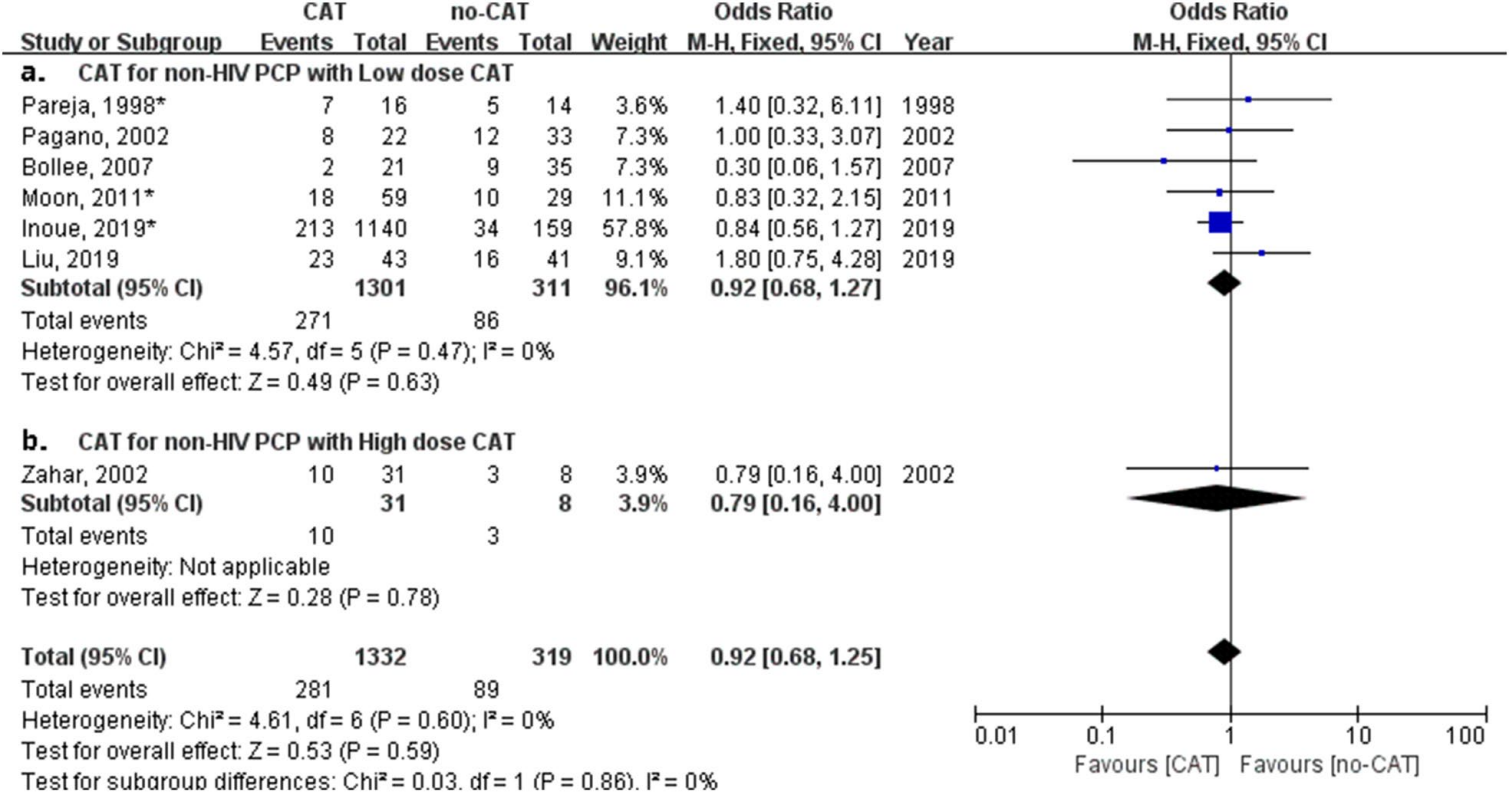
Forest plot for effect of different doses of corticosteroids adjunctive therapy (CAT) on mortality of all non-HIV PCP patients

High dose (> 240 mg prednisone equivalent) or low dose (1 mg/kg/day prednisone equivalent) CAT were reported in 7 studies in non-HIV PCP patients. We performed another meta-analysis on mortality associated with or without CAT in different CAT doses. Figure 5 shows CAT dose had no relation with a lower mortality for non-HIV PCP patients (odds ratio, 0.92; 95% confidence interval 0.68–1.25; *P* = 0.59).

**Fig. 6:**
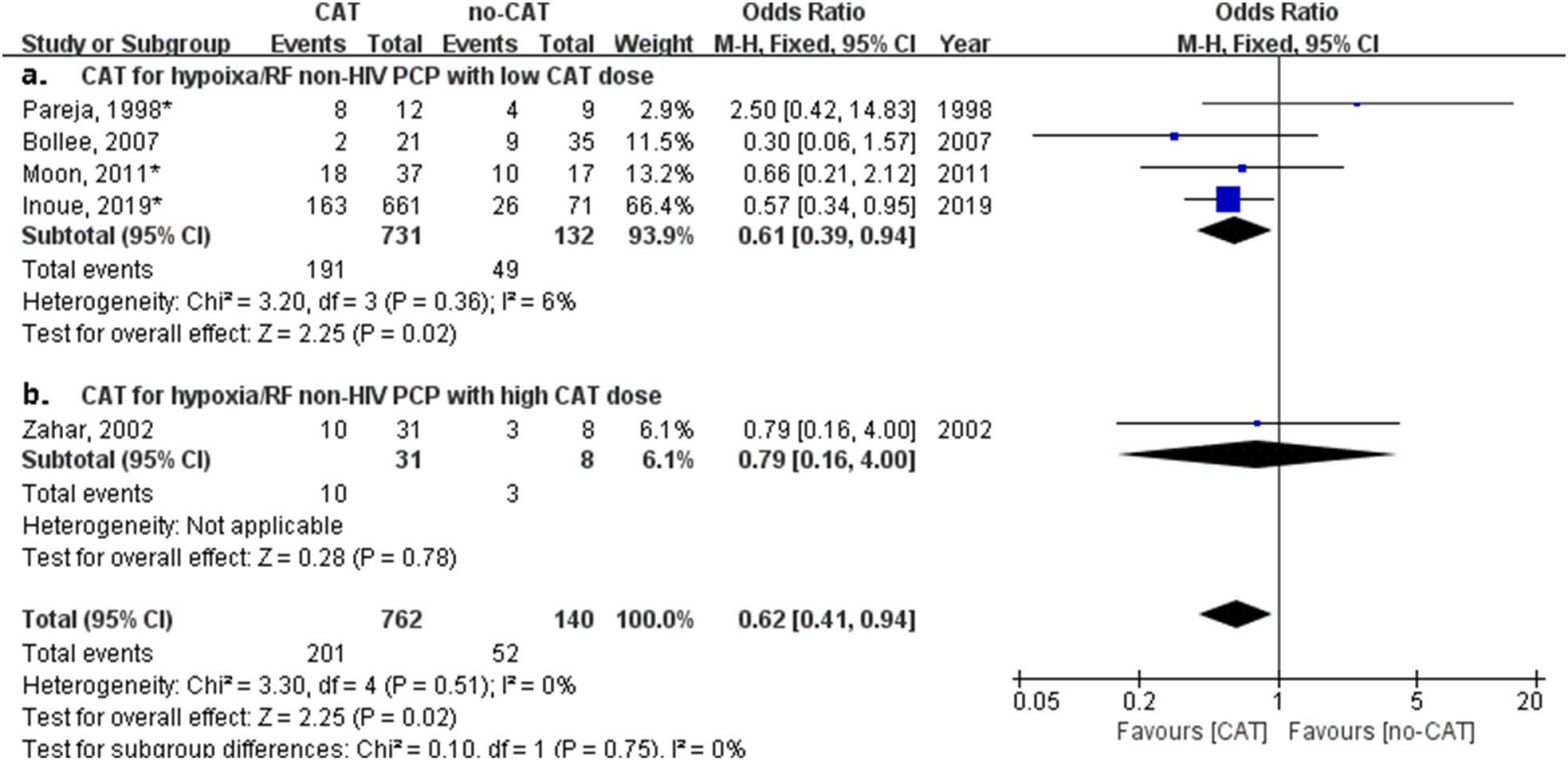
Forest plot of subgroup analysis for effect of different doses of corticosteroids adjunctive therapy (CAT) on mortality of non-HIV PCP with hypoxemia or respiratory failure

Subgroup analysis for hypoxemia patients with high or low dose CAT were performed (as shown in Fig. 6). Treatment with low dose CAT for non-HIV PCP patients with hypoxemia had a significant impact on mortality (odds ratio, 0.61; 95% confidence interval, 0.39–0.94; *P* = 0.02, Fig. 6a). Treatment with high dose CAT for non-HIV PCP patients with hypoxemia had no significant impact on mortality (odds ratio, 0.79; 95% confidence interval 0.16–4.00; *P* = 0.78, Fig. 6b). Low dose CAT was related to a significantly lower mortality for non-HIV PCP patients with hypoxemia.

### Subgroup analysis of studies published before or after 2005

Based on over 20 years of publication range (from 1998 to 2019) of included studies, all studies published after 2005 had an inclusion period after 2000, which may represent different ventilation strategies (lung protective ventilation for ARDS) applied to severe PCP pneumonia induced respiratory failure. Subgroup analysis for the impact of publication range on studies was performed.

**Fig. 7:**
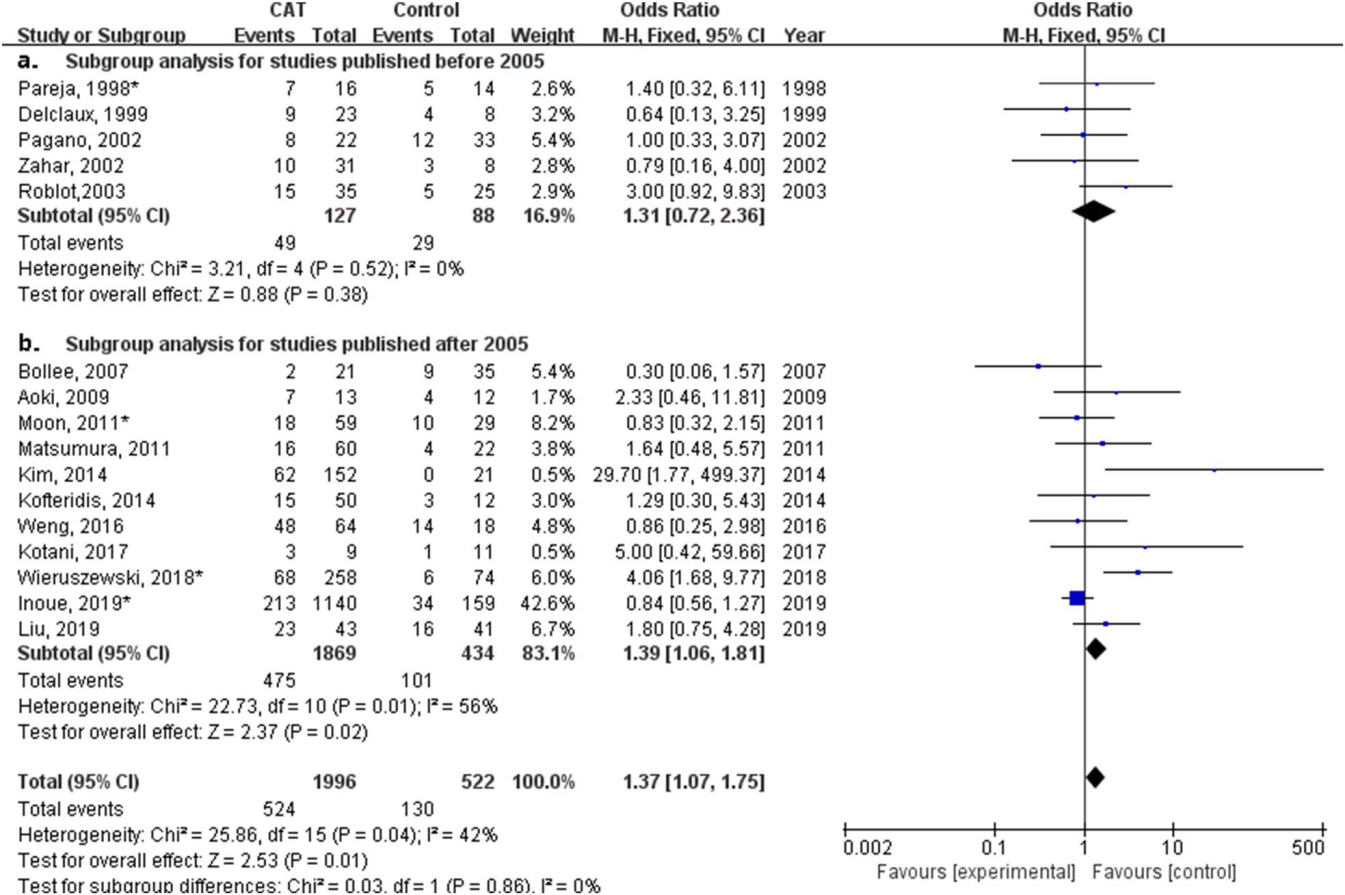
Forest plot of subgroup analysis of studies published before and after 2005 for effect of different doses of corticosteroids adjunctive therapy (CAT) on mortality of all non-HIV PCP patients

In subgroup analysis of 11 studies published after 2005, treatment with CAT for non-HIV PCP patients had a significant impact on mortality (odds ratio, 1.39; 95% confidence interval 1.07–1.75; *P* = 0.01, Fig. 7b). In subgroup analysis of five studies with hypoxemia or respiratory failure published after 2005, Fig. 8b shows CAT was related to a significantly lower mortality for non-HIV PCP patients with hypoxemia (odds ratio, 0.64; 95% confidence interval 0.42–0.96; *P* = 0.03).

**Fig. 8:**
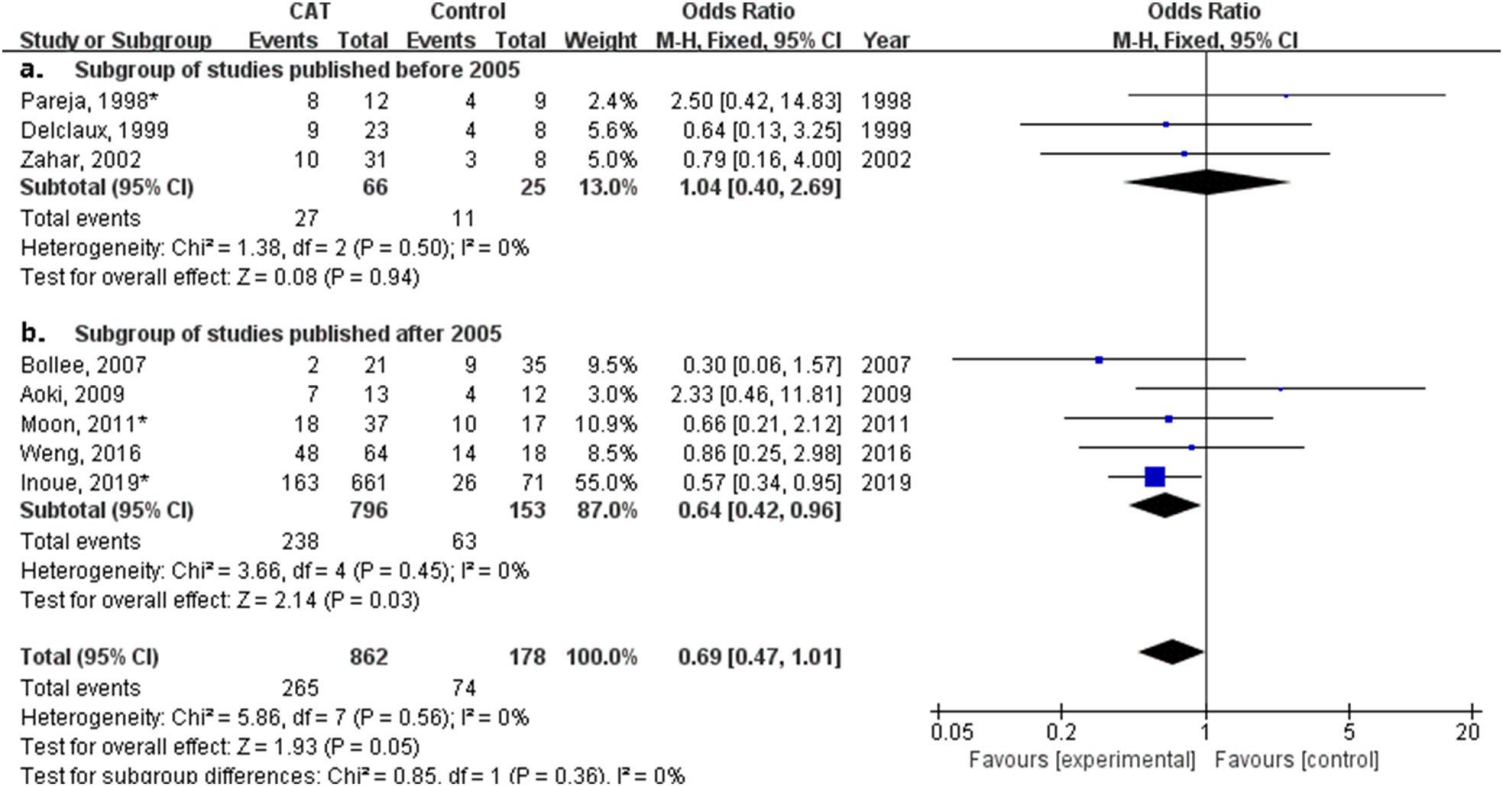
Forest plot of subgroup analysis of studies before and after 2005 for effect of different doses of corticosteroids adjunctive therapy (CAT) on mortality of non-HIV PCP patients with hypoxemia or respiratory failure

### Publication bias

More than eight studies reported mortality in all studies and in studies with hypoxemia patients, so we used funnel plot analysis to assess publication bias. As shown in Additional file 2, there was no obvious evidence of publication bias for mortality in all studies and in studies with hypoxemia patients by funnel plots.

## Discussion

Our systematic review and meta-analysis revealed for the first time that CAT is harmful in unselected non-HIV PCP patients with or without respiratory failure, but may reduce mortality in the subgroup of non-HIV PCP patients who have acute hypoxemic respiratory failure. The main strength of our study is that it had the largest sample size of 2518 cases from 16 studies of non-HIV PCP patients treated with or without CAT.

Firstly, our analysis found that when evaluated in the whole non-HIV PCP population, CAT showed an association with higher mortality. This result indicated that CAT may increase the mortality in unselected PCP patients, including those with or without hypoxemia. This finding is different from that in the HIV-infected population. In a large RCT for HIV-PCP [7], clinical benefit of early CAT could not be demonstrated for HIV-infected patients with mild hypoxemia (a partial pressure of oxygen greater than 75 mmHg on room air), but CAT also showed no harm for mortality. In the most recent two large sample studies in non-HIV PCP, there was no difference in mortality between CAT and no CAT [4, 5].

Secondly, our analysis found a probable association between CAT and low mortality in non-HIV PCP patients with hypoxemia or respiratory failure (OR = 0.69, 95% CI 0.47–1.01, *P* = 0.05). This result is similar to findings from the HIV-infected patients. Prospective studies in hypoxemic HIV patients with PCP found that early initiation of corticosteroids reduced the need for mechanical ventilation and conferred the greatest mortality benefit [6, 7, 24, 25]. Moon et al. [19] evaluated CAT in moderate to severe non-HIV PCP, and also indicated that CAT may not improve the outcome of PCP in non-HIV patients. However, when considering the heterogeneity of the present analysis (*I*^2^ = 47%), the different treatment protocols between studies and the long time elapsed between the different studies can lead to wide differences in the prognosis of critically ill patients, including mechanically ventilated patients. Furthermore, as for the *P* = 0.05, this result could not be considered for a conclusive negative recommendation of CAT use in non-HIV PCP with respiratory insufficiency. The negative result may be due to the design of this analysis, which only included retrospective observational studies, and used different definitions in time and dose of CAT use, which led to heterogenous effects to the outcome.

Thirdly, our analysis found an association between CAT and low mortality in non-HIV PCP patients with respiratory failure(PaO_2_ < 60 mmHg). Limited studies reported the effects of CAT on mortality of non-HIV PCP with respiratory failure. Pareja et al. [9] described that the adjunctive corticosteroids shortened the duration of the mechanical ventilation and may accelerate recovery in cases of non-HIV PCP. Although their study suggested potential benefit of using CAT, it showed no difference in mortality. Lemiale et al. [10] focused on the effect of CAT in non-HIV severe PCP admitted to ICU, and found that high dose steroid was even associated with increased mortality. And recently, Inoue et al. [4] analyzed the severe non-HIV PCP group with and without CAT in a nationwide case series, and revealed that CAT was associated with lower risk of 60-day mortality in non-HIV PCP patients with severe respiratory failure. Moreover, even though a low PaO_2_ (< 60 mmHg) is part of the definition of acute respiratory insufficiency, the use of the level of PaO_2_ alone has some limits to adequately report the severity of *Pneumocystis* pneumonia. The use of PaO_2_/FiO_2_ may be more informative to this purpose. This may have a great impact on the results of the present analysis. The future analysis should consider PaO_2_/FiO_2_ rather than PaO_2_ alone to classify the patients. However, because there were fewer number of studies included, and the standard of the hypoxemia or respiratory failure was subjective based on the retrospective observational studies, the results from the subgroup analysis on hypoxemia and respiratory failure was inconclusive at this stage. Although not definitive, this subgroup analysis at least suggested that CAT may have the potential benefit for mortality in patients with hypoxemia and respiratory failure.

Finally, when we combined the results that CAT increased mortality in unselected non-HIV PCP patients, there was a trend of decreasing mortality in moderate and severe PCP patients with hypoxemia, and significantly decreased mortality in patients with respiratory failure, indicating that CAT may be associated with an increased mortality mainly in PCP without hypoxemia, and with a decreased mortality mainly in severe non-HIV PCP with respiratory failure. These findings is different from that in the HIV-PCP [4, 5, 7]. Therefore, based on our analysis, we speculated that CAT may be avoided in non-HIV PCP without hypoxemia, and may be considered in those with respiratory failure.

Moreover, comparing the impact of high and low doses of corticosteroids is of critical importance. Indeed, corticosteroids are in some patients the factor leading to the development of PCP, and low doses may only represent (both in the physician point of view and by its proper effect) the continuation of the treatment instead of an adjunctive therapy. The classification proposed by Lemiale et al. [10] was very meaningful in terms of the dosage of corticosteroids: no corticosteroids, low dose or high dose. In the study by Inoue and coworkers, patients with respiratory failure had received low dose corticosteroids (mean dose of equivalent prednisone in the present study was 39 mg/day), and they found benefits on mortality with low dose (17–36 mg/day) by subgroup of corticosteroids daily dose, a result quite similar to that proposed in Lemiale’s study.

Our analysis has limitations. First, all studies included were retrospective studies, no prospective randomized trials were included. The studies included used different definitions in CAT and type of mortality, which led to heterogenous effects on the outcome. Moreover, this meta-analysis was performed on retrospective studies, and timing and dose of steroid in severe patients were not clearly described. However, we did subgroup analysis to explore potential heterogeneity. Second, due to lack of reported data, alternative endpoints other than mortality, such as intubation rate, duration of ventilation, and length of hospital/ICU stay, were unable to be analyzed.

## Conclusions

This systematic review and meta-analysis included 16 studies with 2518 participants, providing an overview of research on CAT for non-HIV PCP. Our meta-analysis suggests that among non-HIV PCP patients with respiratory failure, CAT use may be associated with better clinical outcomes, and it may be associated with increased mortality in unselected non-HIV PCP population. We believe that clinical trials are needed to compare CAT vs no-CAT in non-HIV PCP patients with respiratory failure. Furthermore, CAT use may be withheld in non-HIV PCP patients without hypoxemia.

## Supplementary information

**Additional file 1: Figure 2.2.** Subgroup analysis for diferent types of mortality.

**Additional file 2.** Result of publication bias.

## Data Availability

The data can be available in the online database.

## Abbreviations

ICU: Intensive care unit
RF: Respiratory failure
CAT: Corticosteroids adjunctive therapy
PCP: *Pneumocystis jirovecii* pneumonia
