# Supplementary figures and images for: Adjunctive corticosteroids may be associated with better outcome for non-HIV Pneumocystis pneumonia with respiratory failure: a systemic review and meta-analysis of observational studies
Source: Ann Intensive Care. 2020 Mar 20;10:34. doi: 10.1186/s13613-020-00649-9 (PMC7083987; doi:10.1186/s13613-020-00649-9)

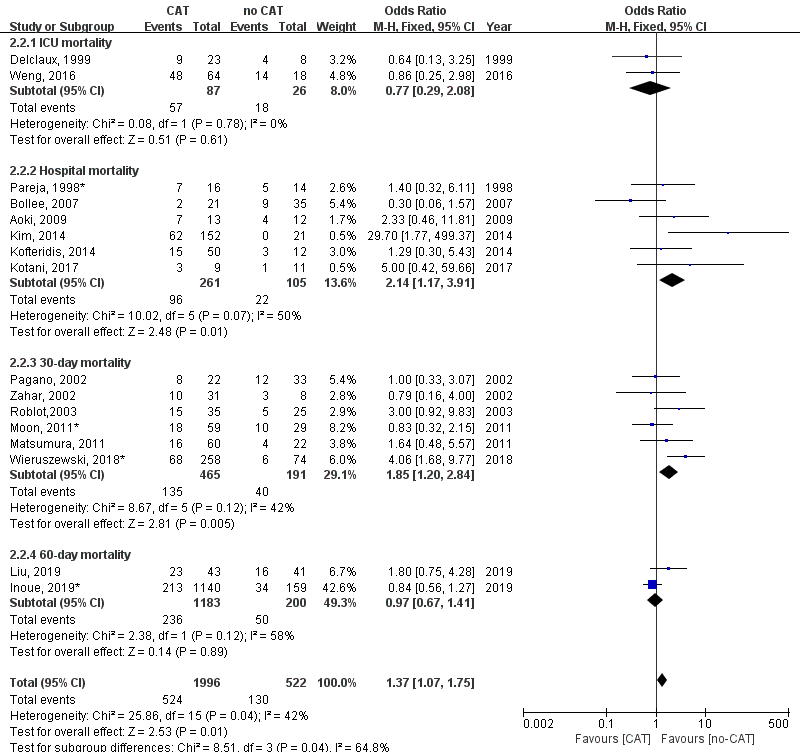

Supplement: Supplementary file 1 — Additional file 1: Figure 2.2. Subgroup analysis for diferent types of mortality. [file 13613_2020_649_MOESM1_ESM.png]
