# Supplementary material for: Adjunctive corticosteroids may be associated with better outcome for non-HIV Pneumocystis pneumonia with respiratory failure: a systemic review and meta-analysis of observational studies
Source: Ann Intensive Care. 2020 Mar 20;10:34. doi: 10.1186/s13613-020-00649-9 (PMC7083987; doi:10.1186/s13613-020-00649-9)

Figure S1. Funnel plot for CAT on mortality in non-HIV PCP patients.


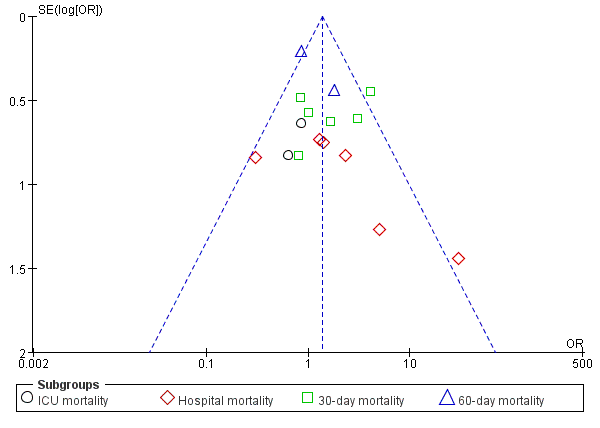


Figure S2. Funnel plot for CAT on mortality in non-HIV PCP patients with hypoxia or respiratory failure.


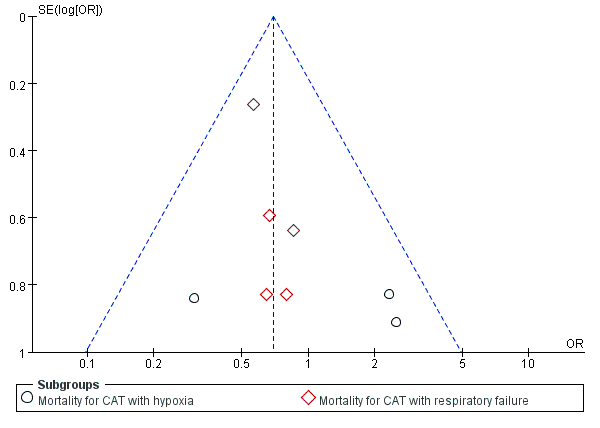

Supplement: Supplementary file 2 — Additional file 2. Result of publication bias. [file 13613_2020_649_MOESM2_ESM.docx]
